# Letter to the editor

**DOI:** 10.1007/s00167-022-07178-x

**Published:** 2022-10-07

**Authors:** Paweł Bąkowski, Tomasz Piontek

**Affiliations:** 1 Rehasport Clinic, ul. Górecka 30, 60-201 Poznań, Poland; 2 Department of Spine Disorders and Pediatric Orthopedics, University of Medical Sciences Poznań, Poznań, Poland

Dear Editor,

we have read recently published article, entitled Endoscopically assisted reconstruction of chronic Achilles tendon ruptures and re‐ruptures using a semitendinosus autograft is a viable alternative to pre‐existing techniques by Nilsson et al. [1] with interest. However, as experts in the field of Achilles tendon reconstructions, we felt obligated to express our concerns about this publication.

First, the technique presented by Nilsson et al. is not novel. Endoscopically assisted Achilles tendon reconstruction with hamstring graft was described in 2016 [2]. In the Nilsson’s technique, there is only one technical difference when compared with the previous technique, namely the Authors use only one tendon graft, instead of two. Moreover, the technique presented by our team in 2016 [2] was further validated with 12-month outcomes, as published in KSSTA in 2020 [3]. This publication was not noticed nor cited by the Authors.

In our opinion, changing only one parameter in the existing and validated technique (that is using just one graft instead of two) and not comparing the outcomes with the outcomes of the existing technique is insufficient to name the technique “a viable alternative to pre‐existing techniques”.

Additionally, below we have listed some general concerns to the study:

1. In the presented surgical technique, the Authors do not show skin incisions in details. They describe the technique as “a mini-invasive with less skin opening” but in fact they use 5 cm skin incision in the proximal region. Moreover, the distal skin incision in the heel bump region is, based on our observations, the key to limit wound healing problems. In our opinion, Authors should show in details how they manage this step.
2. Distal graft attachment is not anatomic. Authors did not take it into consideration in the discussion section.
3. The postoperative rehabilitation protocol presented by Authors is conservative. Patients with chronic Achilles tendon ruptures have a lot of functional limitations and muscle weakness, sometimes they even develop muscle hypotrophy. Prolonged cast immobilization may further lead to intensification of these problems. In our study from 2020 [3], we performed cadaver biomechanical testing, which led to a prominent change in the rehabilitation program. With specific graft stabilization, the patients are able to walk without crutches from day 1 after the surgery.
4. Complications: two out of 22 patients in the presented study [1] were treated with oral antibiotics. Does it mean that they had wound infection? To our knowledge, a key area in this type of procedure is the distal attachment site; a smaller cut in the skin can lead to skin irritation when drilling the heel.

At the end of this letter, we would like to emphasize once again that presented by Nilsson et al. [1] technique is of special interest in the field. As orthopedic surgeons we think that better understanding of the Achilles tendon treatment is of crucial importance in the future and we are staying open to discussing the technical issues.
